# Properties of Luffa Cylindrica Mats Reinforced Castor Oil-Based Polyurethane Composite as an Alternative for Oriented Strand Board

**DOI:** 10.3390/polym14245533

**Published:** 2022-12-17

**Authors:** Anna Carolina Cerqueira Neves, Felipe Perissé Duarte Lopes, Noan Tonini Simonassi, Carlos Maurício Fontes Vieira, Sergio Neves Monteiro

**Affiliations:** Advanced Materials Laboratory (LAMAV), State University of Northern Rio de Janeiro (UENF), Campos dos Goytacazes 28013-602, Rio de Janeiro, Brazil

**Keywords:** luffa cylindrica, composite, fiberboard, castor oil polyurethane

## Abstract

The main objective of this work was to produce and characterize a novel ecofriendly castor oil-based polyurethane (COPU) matrix composite reinforced by Luffa cylindrica mats, luffa for short, to be used as panels, as an alternative to oriented strand board (OSB). To do so, the mechanical behavior was evaluated by tree point flexural, perpendicular o surface tensile, screw pullout, and impact tests that were carried on the novel composite along with the neat matrix. Furthermore, the physical characteristics, the thermomechanical behavior, and the functional groups of the materials were observed by water absorption and thickness swelling tests along with dilatometry and Fourier transform infrared spectroscopy (FTIR). A comparison with commercialized OSB was also performed for control. The luffa/COPU composite was prepared by hand lay-up with 48 vol% of luffa mats incorporated as the maximum allowed by the mold under the available resources for manufacturing. The luffa fibers acted as a good reinforcement for the COPU matrix, where the flexural strength and modulus of elasticity were increased by more than 23 and 10 times, respectively, and the other mechanical properties more than doubled for the composites compared to the neat COPU resin. In general, the composite presented a lower performance compared to the commercial OSB, with the impact results being the exception. The water absorption and thickness swallowing results showed an already-expected behavior for the studied materials, where the better performance was found for the hydrophobic neat resin. The FTIR revealed that there was little interaction between luffa and COPU resin, which can be translated to a weak interface between these materials. However, the mechanical behavior, together with the other results presented by the luffa/COPU composite, confirm it is more than enough to be used as civil construction panels such as OSB.

## 1. Introduction

The use of synthetic fibers has recently been questioned as they are not environmentally-friendly materials, either due to their form of disposal or the high consumption of energy during their manufacturing [1,2,3]. Based on the growing concern regarding sustainability, a tendency has been observed for the replacement of synthetic fibers with natural lignocellulosic fibers (NLFs) which are less harmful to the environment [4,5,6]. Indeed, NLFs have the advantage of being extracted from renewable sources [7,8]. Moreover, NLFs are associated with a relatively high specific mechanical strength, modulus of elasticity, low weight, and low cost, as well as good acoustic and thermal properties [9,10,11]. This justifies the industrial application of polymeric matrix composites reinforced by NLFs [12], especially in the automobile [13,14], and aerospace industries, [15,16] as well as in civil construction [17,18] and ballistic armors [19,20].

Still regarding the substitution of synthetic materials with more sustainable options, recent studies have not been limited to the composite reinforcement but also to the polymeric matrix. Indeed, alternative biopolymer resins to those derived from petroleum have been suggested [21]. This is the case for castor oil-based polyurethane resin (COPU). Along with its good mechanical properties, the plant-based COPU polymer is a non-toxic biodegradable [22,23,24] resin that can be used as promising reinforcement for composite matrixes [25].

One particular NLF is the *Luffa cylindrica*, luffa for short, also known as sponge gourd. Unlike many NLFs, luffa has intercrossed fibers which form a natural mat. The use of these fibers as composite reinforcement has been reported to significantly improve the mechanical behavior of polymeric matrices [26]. This mat structure, along with the luffa’s naturally low density and good mechanical behavior, justify the amount of studies and applications proposed in the most diverse fields, such as the development of filtering mechanisms [27] and sound insulation [28], as well as other civil industry [29] applications.

As for the specific use of COPU matrix composites reinforced with luffa mats to obtain conventional oriented strand board (OSB) panels, no scientific or industrial records were found, which enhances the importance of the present investigation. Although luffa mats can be processed into chips such as those used in the production of OSB, the use of this material as mats would produce a more continuous and cost-effective plate. In order to classify the use of luffa/COPU composite as OSB, or classify it as different types of panels such as high and medium density fiberboard (HDF and MDF), tree standards should be attended, specifically the ABNT NBR14810 [30], EN300 [31] and ANSI A208.1 [32] standards.

Based on these considerations, this work aims, for the first time, to comparatively study the mechanical behavior of a commercial OSB, a plain COPU, and a COPU matrix composite reinforced with luffa mats. The main objective of this study is to find sustainable natural materials that represent both viable technical and environmental alternatives for the civil industry applications. Finally, the novelty of this work was the production of a composite with more than 80 % vol derived from a renewable green origin that presented a general performance for the proposed application since there is a small amount of work of significance proposing composites made by both phases of natural origin [33,34].

## 2. Materials and Methods

### 2.1. Materials

The COPU resin AGT 1315 was supplied by Imperveg company, from Aguaí-SP, Brazil. The COPU resin is a bi-component, 100% solid (solvent free), formulated by cold mixing a prepolymer (component A) and a polyol (component B), both liquid before the mixture, with a gelation time around 15 min; it became hard to the touch after 60–90 min.

**Figure 1:**
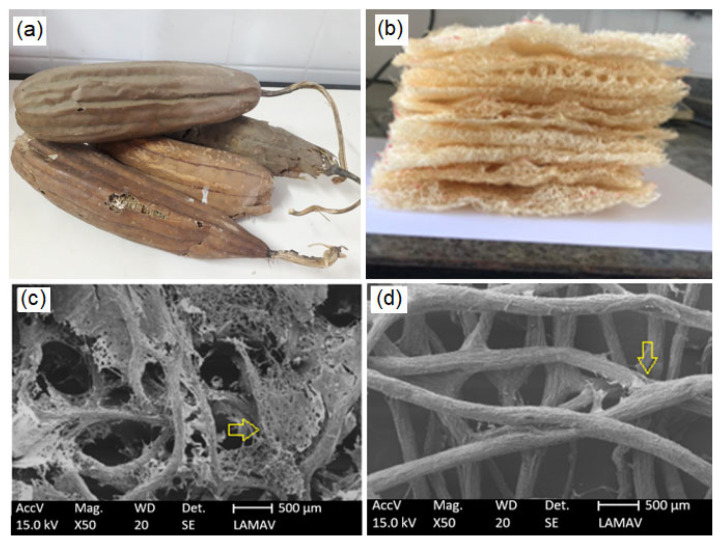
Naturally-occurring luffa sponge gourd as received (**a**) and after the peeling off process (**b**), and SEMs photomicrographs of the luffa mat surface before (**c**) and after the cleaning process (**d**). The arrows point to some of the sites where the fibers are naturally bonded together, forming a mat.

The luffa sponge gourd, illustrated in Figure 1a, was provided by a farmer producer based in Cambuci, Brazil. The commercial OSB used as reference was purchased in the local market of Rio de Janeiro, Brazil.

The dry commercial OSB used as reference was purchased in a local market of Rio de Janeiro, Brazil and was made by a Brazilian manufacturer (LP Brasil) with chip sizes around 5 × 2 cm, with 10 mm thickness and 2.20 × 1.22 dimensions.

### 2.2. Methods

#### 2.2.1. Composite Preparation

Luffa fiber mats shown in Figure 1b were obtained by peeling off the gourd (Figure 1a) and washing it in running water in order to extract surface waxes and other impurities that can make a good interaction between the fiber and resin difficult. Figure 1c,d shows the difference between the fiber before and after the washing process.

The luffa mats mass was constantly measured over time and after 24 h at 60 ℃ there was no weight variation observed, and the moisture content on the fiber surface was considered at a minimum value. The temperature of 60 ℃ was selected in order to avoid excessive fiber degradation. In order to avoid excessive surface moisture and enhance the interaction between the fiber and the matrix, the luffa fabric was mixed to the COPU polymer while still hot.

Since the luffa fibers are a biodegradable material, there is concern regarding the durability of such fibers. The cleaning and drying process not only enhances the quality of the fiber/matrix interface, but will also make it difficult for fungi and bacteria to spread on the surface of the fiber once it is inserted on the polymer matrix, enhancing its durability.

In order to mimic the aspect of OSB panels, the luffa/COPU composites were prepared as short plates in a metallic mold using the hand lay-up technique associated with pressure. The luffa mats were impregnated by the still liquid bi-component resin mixture in a 1.0:1.8 proportion of pre-polymer and polyol, respectively, inside the mold. Then, the fiber and COPU mixture was left inside the closed mold under a pressure of 10 tons in a hydraulic for 24 h at room temperature (RT) until the COPU resin curing process was finished.

The COPU resin presents a low volume variation after the gelation point according to the manufacturer and, since most of the pressure applied to the composite was directly dispersed by the mold, the use of such level of pressure for 24 h ensured a better interaction between the fiber and the liquid resin that could minimally enhance the interface. The composite plates were unmolded and laid to rest for 72 h, following the manufacturer’s recommendation for complete cure of the COPU matrix. Neat resin plates were also made by this method.

The luffa fiber density was obtained as 0.78 ± 0.02 g/cm^3^ by picnometry with distilled water in a 50 mL pycnometer. Since the luffa mat presents relatively large gaps between the fibers, as shown in Figure 1c, the total amount of reinforced material that can be inserted on the composite is limited. In fact, the total amount that could be incorporated as reinforcement in the COPU matrix was found to be 18 layers of luffa mat. The amount of luffa mat incorporated on this novel luffa/COPU composite was approximately 50 vol%.

One goal of this work was to produce a composite with the highest amount of luffa reinforcement and, later, study its possible uses as an OSB. Therefore, each result obtained is discussed regarding the ABNT NBR14810 [30], EN300 [31], and ANSI A208.1 [32] standards.

The metallic mold used had internal dimensions of 150 × 120 mm and its thickness could be varied up to 12 mm by using specific shims. Therefore, the composite plates were produced with a constant size of 150 × 120 mm while its thickness was previously selected according to the standard requirement of each performed test. Finally, samples were cut from the plates with each respective required standard size.

#### 2.2.2. Flexural Tests

In order to determine the flexural strength and modulus of elasticity, three-points static bending tests were carried out as per ASTM D790 [35] in a universal testing machine model 5520, Instron, USA. For each material tested, nine samples were prepared with 120 × 15 × 6 mm and subjected to tests at a 2 mm/min deformation rate and a 90 mm gap between supports.

The tests were conducted until fracture occurred or a 100% deflection was observed. The flexural strength and flexural modulus were calculated using the standard equations [35].

#### 2.2.3. Tensile Strength Perpendicular to Surface

Aiming to investigate the cohesion of the panels layers, tensile tests perpendicular to plates surface were performed in the same Instron 5520 machine according to the ASTM D1037 [36] standard in 10 samples with 50 × 50 × 12 mm dimensions for the novel luffa/COPU composites, the plain resin, and the commercial OSB. The internal adhesion strength of the panels was calculated by the maximum load provided by the tensile test divided by the surface area as indicated by the standard [36]. According to the standard, a high shear performance glue was used to attach the samples on metallic plates in a sandwich-like sample. The sample was subjected to tensile tests with a 2 mm/min crosshead speed until ruptured in a controlled RT of 25 ℃. The test was considered acceptable [36] if a separation of matrix/reinforcement occur. On the contrary, the test was repeated if a fracture occured on the glue.

#### 2.2.4. Screw Pullout Tests

Screw pullout tests were also based on the ASTM D1037 standard [36] for wood-based fiber and particle panel materials. These were performed in the same Instron 5520 machine in a controlled temperature of 25 ℃. For the sample preparation, it was necessary to insert a screw in the center of the plates with 70 × 50 × 12 mm, which were then fixed in the testing machine. Each material was studied used 10 samples on this test. Following the standard [36], a 2.8 mm hole was made through each sample and a 3 mm thick screw was used. The screw pullout resistance was read as the maximum load (N).

#### 2.2.5. Impact Test

Impact tests were performed using both Charpy and Izod settings according to ASTM D6110 [37] and D256 [38], respectively. The tests were conducted at RT in a universal impact test machine model XC-50, manufactured by Pantec, Brazil. Seven samples of each type of material investigated were tested. The results obtained were statistically analyzed and the impact energy absorption of the materials was accessed.

According to the standard [37], the plates were cut with dimensions of 120 × 12 × 12 mm for the Charpy tests and 60 × 12 × 12 mm for the Izod ones [38], and a 45° with 2 mm deep notch was machined on each sample. The fracture surfaces of the impact samples were analyzed by scanning electron microscopy (SEM) on a microscope model SSX 550, Shimadzu, Japan.

#### 2.2.6. Water Absorption and Thickness Swelling

In order to evaluate the materials resistance in a high humidity atmosphere, the water absorption and swelling were analyzed. The procedure for determining water absorption and thickness swelling was also based on the ASTM D1037 standard [36]. Ten samples of each investigated material were water-submersed in periods of 2 and 24 h. After each period, the samples were superficially dried with a cotton cloth and had their thickness and weight measured. The calculation of the moisture content was based on weight differences after the immersing periods. The swelling was based on the samples thickness differences after the immersing periods. Both water absorption and thickness swelling are expressed in terms of percentage.

#### 2.2.7. Thermomechanical Analysis (TMA)

TMA was carried out on the plain COPU resin and the luffa/COPU composite in a dilatometer model DIL 402 PC, Netzsch, Germany, according to the ASTM E228 standard [39], using samples with dimensions of 8 × 13 × 7 mm at a heating rate of 0.5 ℃/min until 200 ℃.

#### 2.2.8. Fourier Transform Infrared Spectroscopy

Fourier transform infrared (FTIR) spectroscopy was used to study the functional groups present in the materials according to the ASTM D5477-18 standard [40]. For this analysis, the samples were dried on a stove at 60 ℃ for 24h and later ground into a powder and added on a KBr paste. This mixture was pressured until a 1 mm thickness was obtained. The tests were carried out on a spectrometer model IRAffinity-1, Shimadzu, Japan, with a 4 cm^−1^ resolution and 32 scans on a wavenumber varying from 4000 to 400 cm^−1^ at a 27 ℃ controlled temperature.

## 3. Results and Discussion

### 3.1. Flexural Strength

**Figure 2:**
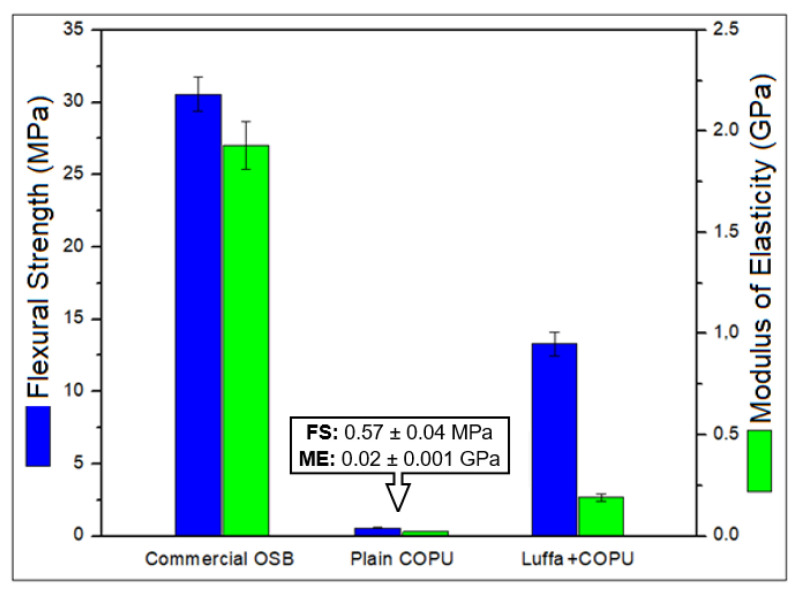
Flexural strength (FS) and flexural modulus of elasticity (ME) properties for the tested materials.

The values of flexural strength (FS) and the modulus of elasticity (ME) for the three tested materials are shown in Figure 2. This figure shows that the luffa/COPU composite presented 13.3 ± 0.8 MPa for FS and 0.19 ± 0.02 GPa for ME. When analyzing the data obtained regarding the flexural properties of the materials tested, it should be noted that, due to the low performance of the plain COPU resin, the final composite, despite being 23 times more resistant to bending with the addition of luffa mat, still displayed a 50% lower performance compared to the commercial OSB. This indicates that luffa is a potentially useful reinforcement material.

By using epoxy as a matrix, the flexural strength can vary from around 20 [41] up to around 55 [30] MPa depending on factors such as the volume of luffa used and surface treatment, among other factors. In fact, according to Mohanta and Acharya [41], the FS of the epoxy matrix composites can be enhanced by almost 60% simply by adding two layers of luffa, while the ME can be enhanced up to 40% higher than the plain resin. As for the surface treatment, Mohanta and Acharya [42] have shown that benzoyl-treated luffa improved an epoxy matrix composites flexural strength by more than 50 MPa. This is a value almost 70% higher than that of the untreated fibers, and the same proportion was observed on the value of ME that varies from 2 up to 3.5 GPa. By using a poly (lactic) acid (PLA) biodegradable resin, Patra, et al. [43] found similar results to those found for the epoxy resin ME, with a FS which was higher for luffa fibers irradiated by electron beam.

In all of these cases [41,42,43], the luffa fibers promoted a substantial enhancement in the mechanical properties of the polymeric resins. The same is true for the novel luffa/COPU composite studied in this work, in which the FS was more than 20 times and the ME was 10 times higher than those of the plain COPU resin.

These results show a good potential for the luffa to be used as polymer matrix composite reinforcement. Differently from man-made fabrics, natural luffa mats occur by the fibers being strongly bonded between each other, as shown in Figure 1. The bonding takes place in many different points on the fiber length, and a relatively large amounts of layers are bonded together. In order to rupture the composite, both the matrix and the interconnections of the fiber mat need to be completely fractured. This is a good anchor mechanism, especially for composites with a weak interface between reinforcement, such as this novel composite and matrix or even low performance matrices such as the COPU resin.

Indeed, NLF-reinforced polymer composites are well known for their weak interface due to the general hydrophobic characteristic of the polymer matrix and the hydrophilic characteristic of the fibers. This is the case for the luffa/Polyurethane composites, where the weak interfaces between the phases were reported together with the mechanical behavior effect of the fiber surface treatment [43,44,45].

Regarding the use of these composites as OSB, according to the NBR14810 standard [30] and considering the flexural properties, they can be used as panels, but not for structural purposes. This is limited to internal use in dry conditions (between 6 and 10 mm in thickness) since the values of FS and ME, for this panel material classification class, are 13 MPa and 0.19 GPa, respectively. Moreover, according to the parameters of the EN300 standard [31], a material can be classified as OSB Type 3 (for 10 mm thickness), where a minimum of 11 MPa and 0.14 GPa is expected for FS and ME, respectively. Finally, for the ANSI A208.1 standard [33], the composite can be classified as an MS category panel for commercial use. For this application, a minimum FS of 12.5 MPa and a minimum ME of 0.19 GPa are required.

### 3.2. Perpendicular to Surface Tensile Strength

**Figure 3:**
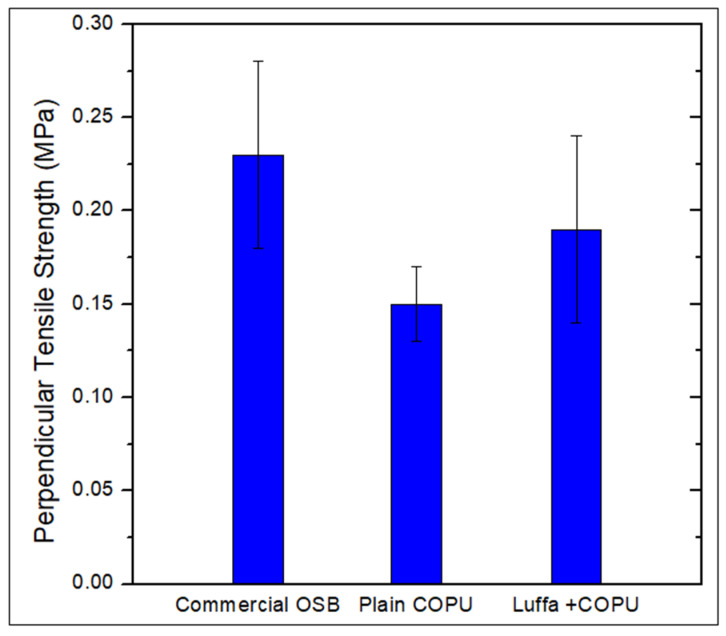
Perpendicular tensile testing results.

Regarding the perpendicular tensile tests for each analyzed material, the strengths were calculated and are shown in Figure 3. It can be seen from the results that the commercial OSB presented superior properties in relation to the luffa/COPU composite studied in this work. This result may be associated with the properties of the adhesive used in making the OBS, indicating superiority in relation to the use of COPU as a matrix.

Regarding the data shown in the Figure 3, there seems to be no statistical difference between the novel luffa/COPU composite and the commercial OSB, even if there is an obvious difference between the plain COPU resin and the commercial OSB. In fact, after the analysis of variance (ANOVA) was applied, the *p*-value which was obtained was greater than the significance of 0.05 between the novel luffa/COPU composite and the commercial OSB. Thus, the results showed that the addition of luffa mats provided, indeed, an enhancement in performance for the plain resin. Finally, both the luffa-reinforced COPU matrix composite and the commercial OSB failed to meet the minimum requirements criteria of any of the three analyzed standards.

The behavior observed on the luffa composite and the COPU resin can be linked to the weak interface between matrix and fiber. However, the better result for the composite can be associated with the luffa mat configuration. As already discussed in this work, in accordance with the literature [43,44,45], this interlink between the luffa fibers acts as a good anchor for the resin. This behavior is most likely responsible for the composites enhanced behavior compared to the plain COPU resin.

### 3.3. Screw Pullout Tests

**Figure 4:**
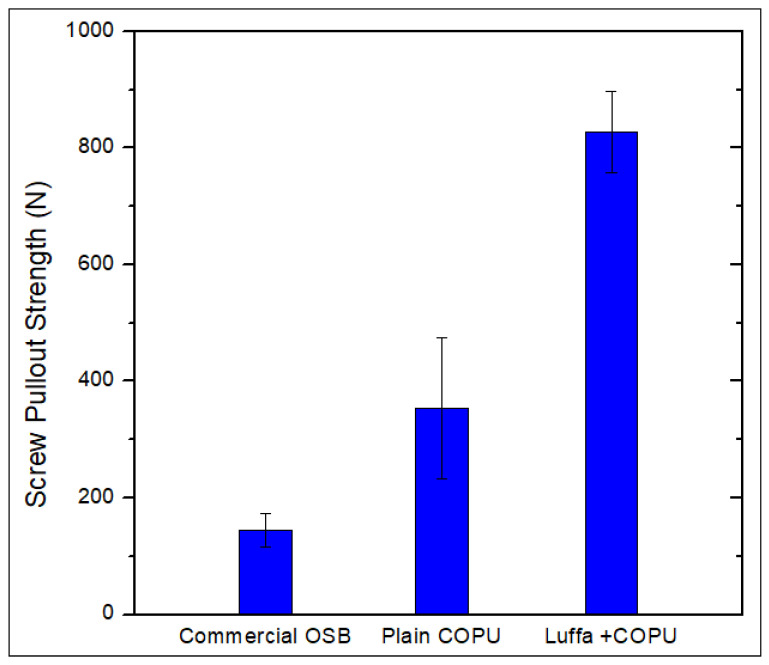
Screw pullout strength results.

The maximum load values of the resistance to screw pullout are shown in Figure 4. The pullout resistance obtained for the luffa/COPU composite was approximately 6 times greater than that obtained for commercial OSB and approximately 2.5 times greater than that obtained for the plain COPU matrix. These values suggest that the incorporation of luffa mat significantly improved the matrix strength, since the fibers in the mat acted as anchor points [42]. Regarding the classification of this novel composite material of COPU matrix reinforced by luffa mat, according to OSB standards [30,31,32], the requirements for resistance to screw pullout appear only in the NBR 14810 standard [30]. The minimum 800 N load value required by this standard is indicated in the case of high-density panels with thicknesses between 14 and 20 mm.

The results presented in Figure 4 affirm the good performance found for the novel developed composite. Not only was the performance better than that of the commercial OSB, but also the luffa/COPU composites presented a main screw pullout strength above the standard requirement even with thinner dimensions. Therefore, it can be affirmed that, for a thinner plate, the composite plate would present performance which is more than satisfactory for plates with double the thickness.

The most likely factor for the enhanced behavior presented by the novel luffa/COPU composite is the interlink between the luffa fibers on the mat. This mat characteristic is responsible for strengthening the matrix, and enhancing its behavior with the luffa addition in contrast to the results presented on Section 3.2 for perpendicular-to-surface tensile tests. Indeed, the main difference between the behavior observed in these tests is that, while the interlink of the luffa holds the composite together on the screw pullout tests, it is irrelevant to hold the mats bonded between each other on the tensile test.

### 3.4. Impact Strength

The impact tests performed in both the Charpy and Izod configurations are presented in Table 1, showing the impact behavior of the commercial OSB, the plain COPU resin, and the luffa/COPU composite. It can be seen that the novel luffa-reinforced composites not only enhanced the matrix impact resistance but also displayed better performance than the commercial OSB on the Charpy and Izod tests.

Regarding the incorporation of luffa in the COPU matrix, it should be noted that there was a significant increase in the toughness resistance. This result was expected, since an increase in the amount of incorporated NLFs as reinforcement in polymer matrices tends to increase the impact energy absorbed compared to their plain matrix [46,47,48]. It is understood that this result is a consequence of a weak interface between the matrix and fiber, resulting from the difference between the polarities of these materials. The natural polar fiber and the non-polar matrix require the system to absorb more energy. This causes rupture, as part of the energy will be spent in the ramifications of the luffa fibers and in the processes of detaching them from the matrix. The luffa’s spongy nature and vascular system also contribute to greater energy absorption [43,44].

**Figure 5:**
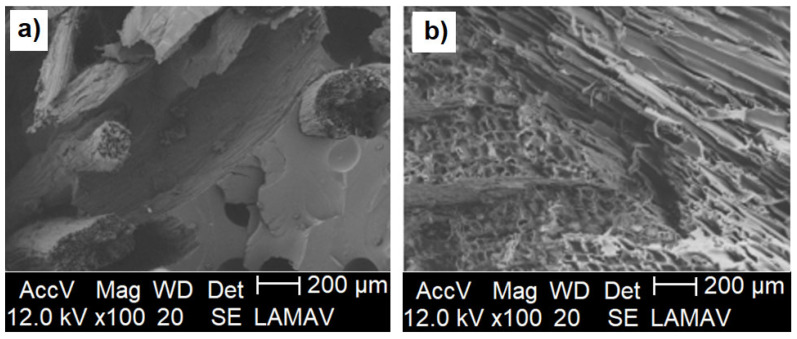
SEM photomicrography of the impact Charpy surface of fracture for the luffa reinforced COPU composite (**a**) and commercial OSB (**b**).

Regarding the surface of fracture, Figure 5 shows that the luffa/COPU composite fractured as well as the commercial OSB. In this figure, the differences between the studied materials structures are shown. While the luffa/COPU composite presents large amounts of resin together with the fibers, the commercial OSB presents the wood chips glued together by a matrix so thin that is almost invisible. Since the luffa fibers are naturally interconnected, the composite material will only fracture if all the fibers are ruptured together, unlike the commercial OSB. This behavior also justifies the results presented in Table 1, in which the luffa/COPU composite presents a greater performance than the commercial OSB for impact and screw pullout tests. The absence of these interconnections between the luffa mats also justifies the relatively lower performance in flexural and perpendicular tensile strength.

### 3.5. Water Absorption and Thickness Swelling

**Figure 6:**
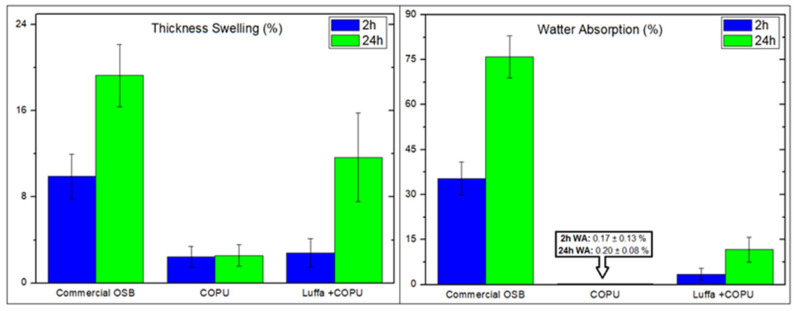
Water absorption (WA) and thickness swelling properties after 2 and 24 h for the commercial OSB, plain COPU, and luffa-reinforced COPU composite.

After immersion of the specimens followed by measurement and weighing at intervals of 2 and 24 h, it was possible to calculate the water absorption rate and the swelling in thickness of the samples as shown in Figure 6. From these data, it is possible to observe that the plain COPU presents negligible water absorption values, in addition to a low swelling rate. Considering that the luffa mats on the composite are embedded by the matrix, the COPU gives the composite greater resistance to water absorption. This does not occur as efficiently with commercial OSB, in which the fragments of wood are not surrounded by a matrix.

Another point that must be considered when observing the significantly higher water absorption rate of the commercial OSB compared to the luffa/COPU composite is that, in the commercial OSB, there is a volume of lignocellulosic material (wood chips) which is greater than in the luffa/COPU composite (Figure 5), which contributes to a higher rate of water absorption and swelling in thickness. This increased swelling ratio with the volume of fibers in the material is reported in the literature [49,50,51,52].

Bera et al. [49] observed, in their epoxy composites reinforced by luffa, that the water absorption of the composite increases with the increase in the volume of fibers, as is expected, since lignocellulosic materials are highly hydrophilic. Jeriwala and Jain [50] concluded that the greater the hygroscopicity of a material, the greater its adverse effects on mechanical properties which, consequently, affects its long-term performance. Miléo et al. [51] reported that the variation in the amount of moisture in the material can also cause a network of microcracks on the surface of the composites, which, in turn, can impair the mechanical properties and also the electrical properties. The authors [51] pointed out that the maximum reported absorption was 40% for polyurethane (PU) composites reinforced by 20 wt% sugarcane bagasse and 20% absorption for the plain PU matrix, a value which is considered high and which can be attributed to the inefficient polymer curing. Jamaluddin et al. [52] reported an improvement in the luffa’s water absorption resistance properties when added to a polyurethane matrix. The neat fiber presented 107.9% absorption against 46.5% in a reinforced PU by a layer of luffa.

Based on the OSB standards reported in the present work, it is possible to classify the existing material. According to the NBR 14810 standard [30], the values found meet the established maximum swelling in 24 h for the panels (12%), once again corroborating its classification as a non-structural panel for indoor use in dry conditions (P2).

As for EN 300 [31], the results also corroborate a classification, which was pointed out when evaluating the material’s flexural properties. Therefore, it is also appointed as an OSB type 3 panel, with the maximum swelling allowed in 24 h of up to 15%.

### 3.6. Thermomechanical Analysis (TMA)

**Figure 7:**
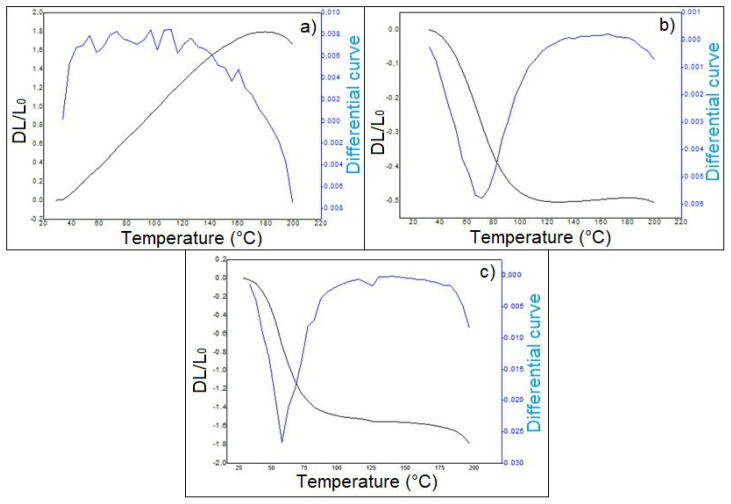
Dilatometry curves for the neat COPU resin (**a**), novel luffa-reinforced composite (**b**), and commercial OSB (**c**).

The influence of the temperature on the materials dimensions is an important behavior that can be used to determine, among other characteristics, the glass transition temperature (Tg) of the polymeric materials [53]. Figure 7 shows the graphical representation for the results obtained by TMA tests carried on the commercial OSB, the neat COPU resin, and the luffa/COPU composite. From 40 up to 100 ℃, a change in behavior occurred: the neat resin had its volume expanded, which was associated with the temperature increase in a shrinking material with the luffa addition. This behavior can be attributed to the already-reported [52] water evaporation that lead to a consequent volume loss in the COPU composites and the commercial OSB, since both are made with hydrophilic lignocellulosic material, as shown in Figure 5.

The TMA tests were conducted up to 200 ℃; therefore, the hemicellulose, lignin, urethane, and polyol degradations, all of which would occur between 255 and 430 ℃ [52], do not appear in Figure 7. However, the peak shown by the neat COPU resin, at 162 ℃, is related to rearrangements of the polymer chains [52] that expanded in almost 2%, as shown in Figure 7a. This rearrangement does not occur as freely on the luffa/COPU composite as it does for the plain COPU resin, most likely due to the luffa mobility restrictions imposed on the polymer chains [54]. Consequently, an expansion no greater than 0.1% was observed for the composites, which means a total expansion more than 20 times lower than that of the plain resin.

The fiber mobility restrictions on the matrix can directly affect the Tg. Usually, the transformation requires a higher amount of energy to occur, as reported by Kalusuraman et al. [47]. They showed a significantly increase in the Tg for epoxy composites reinforced by luffa. Regarding this work, the Tg was obtained as the tangent intersection point before and after the transition [55]. For the neat COPU resin and the luffa/COPU composites, the Tg was found, respectively, to be 38 ℃ and 95 ℃, which is very close to those reported by Jamaluddin et al. [52], who found 49 ℃ for a PU resin and 91 ℃ for the luffa reinforced composites. As for the commercial OSB, the Tg was found to be 76 ℃. This relatively lower value compared to the novel luffa/COPU composites, as well as other epoxy reinforced by natural fiber composites [47], is usually found in materials that absorb water. Indeed, the greater the absorption, the lower the transition temperature.

### 3.7. Fourier Transform Infrared Spectroscopy

**Figure 8:**
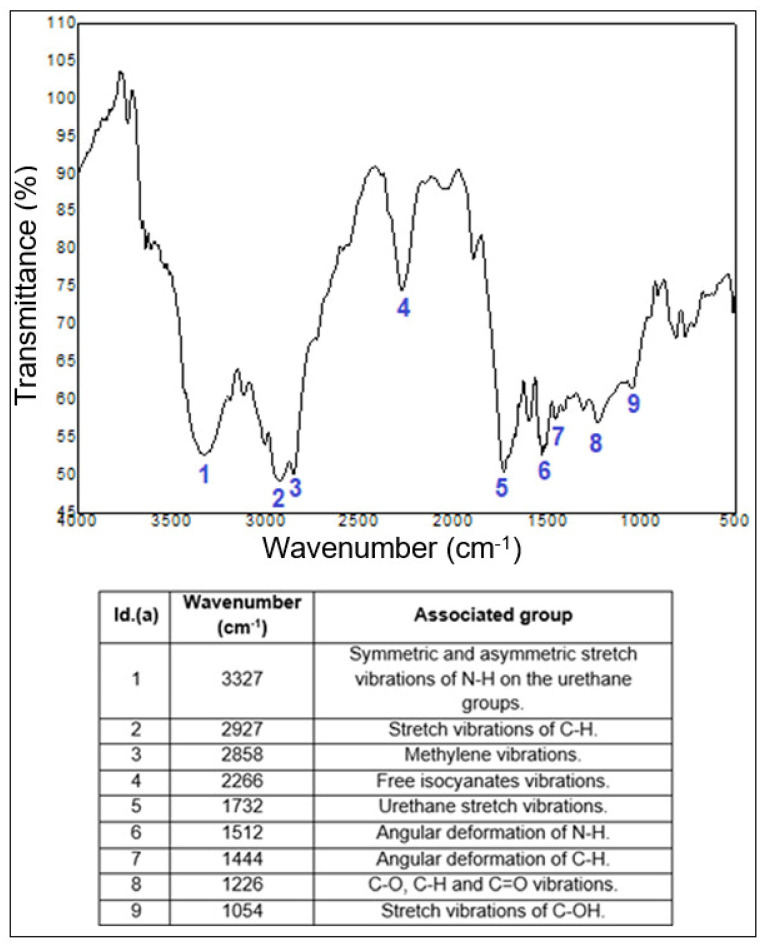
FTIR spectra for the neat COPU resin.

The neat COPU resin, the luffa/COPU composite, and the commercial OSB were subjected to FTIR spectroscopy. In order to facilitate the interpretation of the results, the luffa/COPU composite and the neat COPU resin spectra are presented fist, in Figure 8, which also contains the main identified groups, based on the literature [41,51,54,55,56,57]. The commercial OSB results are presented after the discussion regarding the COPU resin and the luffa/COPU composites in Figure 10.

**Figure 9:**
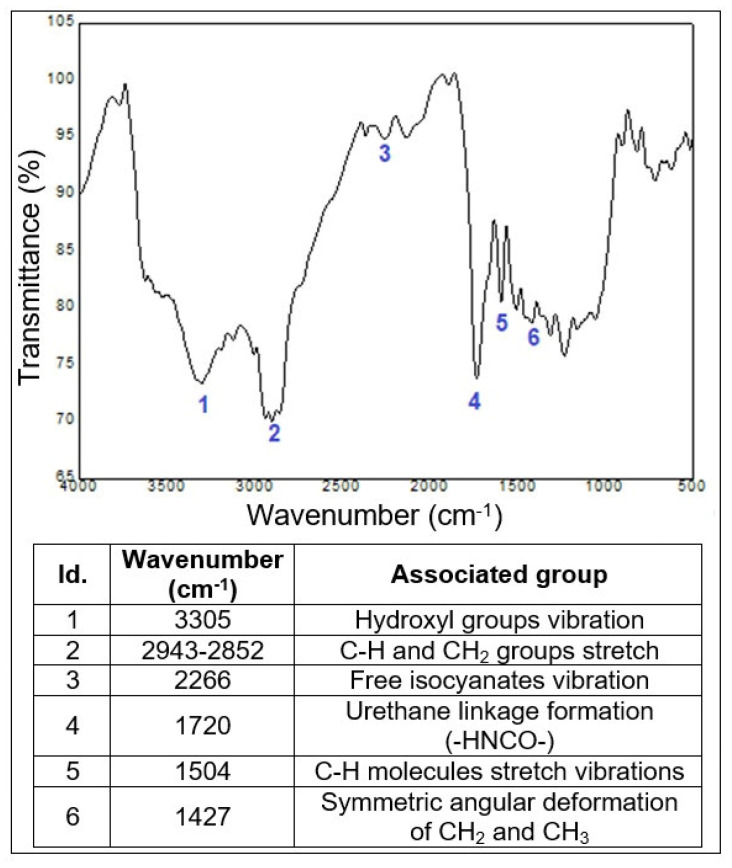
FTIR spectra for the luffa/COPU composite.

**Figure 10:**
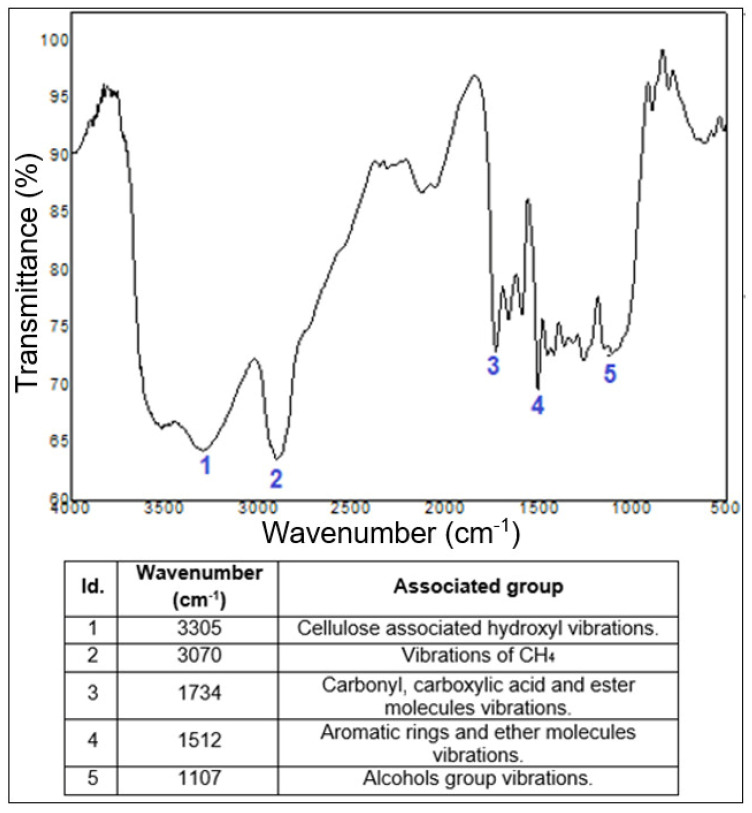
Commercial OSB spectrogram obtained by FTIR.

The free isocyanates (NCO) are reported in the literature to appear around 2270 cm^−1^ [56,57] and their presence is confirmed by the number 4 band at wavenumber 2266 cm^−1^ on the neat COPU resin spectrogram. According to some studies, the NCO groups would react with the -OH of the lignocellulosic fibers to form urethane linkages that are identified at wavenumbers around 1720–1700 cm^−1^ (number 4 on Figure 9) [33,34]. Moreover, the appearance of the free isocyanates band on the composite (band number 3) indicates that the luffa fibers interacted with the COPU, providing a minimum interface strength. Still, the composites did not present a good interface as reported by the literature [44,45], which justifies the performance in the perpendicular tensile strength, and impact and flexural tests performed on the luffa/COPU composite. Finally, all of the other bands expected on both the pure matrix and the composites are shown in Figure 9; these are stretch in the hydrocarbon groups commonly found in PU resin [52,56,57] and the groups found on lignocellulosic materials such as hydroxyls and other functional groups associated with cellulose, hemicellulose, and lignin [41,47,52,58].

It is not common to find the FTIR spectra of commercial OSB and materials to be alike. In fact, most of the studies carried out on such materials are regarding the influence of thermic treatments on the studied material [59,60]. Moreover, it is very hard to detect exactly what kind of polymer is used as matrix for such composites. Indeed, most of the time, the polymers used are actually blends of resins that present good enough mechanical behavior along with low cost, among other technical characteristics [52]. Therefore, the FTIR characterization of this material was limited to identify the wood reinforcement and its components chemical interactions, such as those with cellulose, lignin, hemicellulose, and water. Nevertheless, it is not that difficult to find similarities between the luffa/COPU composite and the commercial OSB spectra, since both the reinforcements are lignocellulosic materials.

## 4. Conclusions

The amount of luffa mat reinforcement possible to be incorporated into castor oil polyurethane (COPU) matrix composite, according to the manufacturing method adopted, was a mass fraction of 39.2%, corresponding to 48 vol% of fiber. It should be highlighted that there was a general increase in the mechanical behavior of the luffa mat-reinforced COPU matrix regardless of the weak interface between the two.

Comparatively, the mechanical properties of the investigated composite were superior to those of both the COPU matrix and a commercial oriented strand board (OSB), except for flexural strength; however, it was still superior to what is required by the OSB standards. Furthermore, considering the other properties, especially the impact resistance, it is possible to assert that luffa mat acts as a reinforcement for the COPU matrix.

This satisfactory result, especially in terms of impact resistance, is related to the luffa natural mat structure that is associated with a weak interface between matrix and fiber. This suggests that the connection between COPU matrix and luffa fiber reinforcement has not occurred due to the weak interface between these materials. The absence of chemical interaction between the composite phases is evidenced by the presence of the free isocyanates groups on both the plain resin and the composites FTIR spectra, which further reinforces the idea of a weak fiber/matrix interface.

Regarding the estimated production cost of the luffa/COPU composite, there are still improvements that need to be made in order to achieve commercial appeal.

As for the OSB standards, in terms of possible applications, it can be concluded that the luffa/COPU composite would be classified as a medium density panel for commercial use according to ANSI A208.1, as a non-structural panel for indoor use in dry conditions P2 by the NBR 14810, and as an OSB panel of the type 3 as per EN300.

## Figures and Tables

**Table 1:** Impact behavior for plain COPU resin, luffa reinforced COPU composite, and commercial OSB.

Test Configuration	Material	Toughness Resistance (J/m)	Impact Resistance (kJ/m^2^)
Izod	Commercial OSB	143.2 ± 21.7	17.3 ± 3.2
	COPU	228.6 ± 13.6	26.3 ± 3.7
	COPU + luffa	379.8 ± 38.5	42.1 ± 7.3
Charpy	Commercial OSB	38.8 ± 6.5	4.4 ± 0.7
	COPU	305.1 ± 3.9	34.7 ± 0.4
	COPU + luffa	380.1 ± 11.4	42.2 ± 1.2
